# Gastroprotective Effects of Aqueous Extracts of Broccoli Stems on Acute Injury in Rats: A Comprehensive Evaluation of Gastric Function and Inflammatory Responses

**DOI:** 10.3390/medicina61010089

**Published:** 2025-01-07

**Authors:** Jihye Choi, Yuseong Jang, Hyeon-Gi Paik, Melissa Hyun-Joo Ha, Jungkee Kwon

**Affiliations:** 1 Department of Laboratory Animal Medicine, College of Veterinary Medicine, Jeonbuk National University, 79 Gobong-ro, Iksan-si 54596, Jeollabuk-do, Republic of Korea; jyyye@naver.com (J.C.); yuseongjang@naver.com (Y.J.); hyeongipaik@naver.com (H.-G.P.); 2 Broccos, Room 503, 3-3 Jongangdae-ro, 296 beon-gil, Dong-gu, Busan 48730, Republic of Korea; broccos.official@gmail.com

**Keywords:** gastroprotection, antioxidants, broccoli extract, cytokines, gastric motility

## Abstract

*Background and Objectives*: Acute gastric injury is a prevalent gastrointestinal disorder characterized by inflammation and damage to the stomach lining. In this study, we investigated the therapeutic potential effects of broccoli stem extract (BSE) against acute gastritis in a rat model. *Materials and Methods*: The antioxidant properties of BSE were evaluated through DPPH and ABTS radical scavenging activity assays and total polyphenol content analysis. Acute gastric injury was induced using 150 mM HCl/60% EtOH, and male SD rats (6-weeks old, *n* = 6/group) were administered BSE by oral gavage at concentrations of 50, 125, and 250 mg/kg. *Results*: The BSE 250 mg/kg group exhibited significant relief of clinical signs compared to the negative control group. In addition, the BSE 250 mg/kg group showed significant improvements in gastric tissue, including macroscopic reductions in ulcer size and improved overall gastric morphology as assessed through gross examination, as well as microscopic improvements such as reduced inflammation and the restoration of mucosal integrity observed in histopathological analysis. BSE modulated NF-κB signaling, decreased inflammatory cytokines (TNF-α, IL-1β, and IL-6), and increased PGE_2_ levels. Pyloric ligation experiments demonstrated reduced pepsin and gastric acid secretion. Improvements in gastric emptying and gastrointestinal motility were also observed in the BSE-treated group. *Conclusions*: These findings highlight the potential of BSE as an effective therapeutic agent for acute gastritis in rats, offering significant improvements in gastric damage, inflammation, and motility.

## 1. Introduction

Peptic ulcers, gastritis, and various digestive disorders are major global health issues, with an estimated 4 million patients experiencing these worldwide each year [1,2]. These conditions contribute to a substantial economic burden, including the healthcare costs and loss of productivity. Although gastrointestinal disorders are not typically fatal, they cause discomfort during daily activities, diminish quality of life, and result in considerable mental distress for patients [3]. The gastric mucosa plays an essential role in maintaining the physiological functions of the stomach. Gastritis occurs due to an imbalance between aggressive factors (such as hydrochloric acid [HCl] or pepsin secretion) and protective factors (such as prostaglandins [PGEs] or antioxidants) [4]. Inflammation of the gastric mucosa is biochemically complex and often driven by an imbalance in reactive oxygen species (ROS) production [5]. Excessive ROS generation is a well-known stressor to gastric epithelial mucosa, activating the NF-κB signaling pathway [6]. This activation increases the levels of pro-inflammatory cytokines such as IL-6 and IL-1β, which contribute to gastric mucosal damage [6,7]. Protective factors like PGE_2_ play a crucial role in counteracting these effects by promoting mucosal repair and reducing inflammation [4]. Evaluating these inflammatory and protective markers is critical for understanding the mechanisms underlying gastritis and potential therapeutic interventions.

Gastritis is characterized by inflammation of the gastric mucosal epithelium and can present with symptoms such as abdominal pain and bleeding [8]. Current treatments for gastritis are primarily based on acid secretion inhibitors, such as proton pump inhibitors (PPIs) or H2-receptor antagonists. These medications function by reducing the production of stomach acid thus providing symptomatic relief and allowing the gastric mucosa to heal [9]. However, reduced gastric acidity can impair protein digestion, leading to the exposure of allergenic proteins in the small intestine and potentially increasing the risk of food allergy development [10]. Furthermore, hypochlorhydria caused by these medications disrupts the bactericidal barrier of the stomach, increasing the risk of bacterial overgrowth and infections [11,12]. Long-term use of these medications is also associated with side effects such as gynecomastia, infertility, osteoporosis, vitamin deficiencies [3], and an increased risk of gastric cancer. Therefore, there is a need to develop natural food-based treatments for gastritis that can be administrated long-term with minimal side effects.

Gastritis induced by non-steroidal anti-inflammatory drugs (NSAIDs), alcohols, and other medications is often accompanied by gastrointestinal disturbances. Symptoms such as abdominal pain and nausea can affect gastric emptying [13]. In particular, patients with autoimmune gastritis were reported to experience delayed gastric emptying compared to those with general functional dyspepsia [14]. Furthermore, since gastric emptying is related to gastrointestinal motility, delayed gastric emptying leads to decreased gastrointestinal motility, which may subsequently affect various metabolic diseases [15].

Cruciferous vegetables such as cabbage, kale, and broccoli have protective properties against gastritis and peptic ulcers. These foods are recognized for their ability to manage various diseases related to inflammation and gastrointestinal disorders [16,17,18,19]. Among cruciferous vegetables, broccoli (*Brassica oleracea* L.) has garnered the most attention due to its abundance of phytochemicals, such as polyphenols and carotenoids [20,21]. These compounds have shown antioxidant [22], anti-inflammatory [23], and anti-carcinogenic effects [24], contributing to their therapeutic potential. Consequently, extensive and diverse research was conducted on broccoli.

Broccoli is a vegetable highly valued for its diverse nutritional benefits, including high levels of antioxidants, vitamins, and dietary fiber [21]. While florets are commonly consumed, broccoli stems, often discarded as waste, also contain bioactive compounds with potential therapeutic effects, such as anti-inflammatory and antioxidant properties. These characteristics make broccoli stems a promising candidate for developing functional food-based treatments for gastritis. Consequently, up to 75% of the stems and remaining parts are discarded, raising ongoing concern about resource wastage [24]. The discarded stems and leaves of broccoli contain 2 to 4 times higher levels of vitamins, β-carotene, and calcium compared to the broccoli flower ball [25]. However, due to their low utilization, these parts are often wasted, leading to environmental pollution such as air contamination, such as carbon monoxide, and particulate matter and water eutrophication [26]. Broccoli stems contain higher levels of malic acid compared to the typically consumed florets and have demonstrated superior DPPH radical scavenging activity at the same concentration [27]. Despite the potential benefits, the underutilization of broccoli stems highlights a significant gap in resource use and environmental sustainability. Addressing this issue could both enhance nutritional intake and mitigate environmental impacts. The aim of this study is to investigate the potential of broccoli stems as a natural treatment for gastritis, focusing on antioxidant effects, and effects of gastric health. This research seeks to provide a sustainable alternative to current pharmacological treatments, with the potential for long-term use and minimal side effects.

## 2. Materials and Methods

### 2.1. Preparation of Broccoli Stem Extract

The broccoli stem extract (BSE) used in this experiment was derived from broccoli purchased from Jeju Island, Republic of Korea. Jeju Island is known for broccoli production during the winter months, and the samples used in this study were harvested between December 2023 and March 2024. The broccoli stems were thoroughly washed under running water to remove dirt and contaminants before being dried. The thin stem parts were dried at 70 °C for 12 h using a hot air dryer. After drying, the stems were ground into power using a grinder. The powder samples (10 g) were mixed with 100 mL of distilled water (DW) at a 1:10 *w*/*v* ratio. Each extraction was performed using fresh DW to ensure efficient compound recovery. Then, they were extracted three times for 1 h each at 60 °C in a water bath. To remove solid particles, the mixture was filtered through NO. 6 filter paper (Advantec Co., Tokyo, Japan). The filtered extracts were concentrated at 40 °C using a vacuum concentrator for approximately 2 h until it reached a concentration of 10–20 brix. The concentrated extract was then freeze-dried at −50 °C under a pressure of 0.01 mbar. The extract was then dried using a freeze dryer. The freeze-dried BSE was stored at −20 °C in airtight containers until further animal study.

### 2.2. Measurement of Antioxidant Capacity

#### 2.2.1. DPPH Radical Scavenging Activity

The DPPH radical scavenging activity of BSE was performed following the methodology outlined in Gulcin et al. [28]. We used ascorbic acid standard material, and BSE was diluted using ethanol to concentrations of 0.5, 1, 2, and 4 mg/mL. The samples and DPPH solution were mixed at a ratio of 1:9, respectively. After thorough mixing, 100 μL of the solution was dispensed into each well of a 96-well plate. The plate was then incubated at room temperature for 10 min in the dark. Upon completion of the reaction, the absorbance was measured at 517 nm using a microplate reader (Synergy 2, BioTek Instruments, Winooski, VT, USA).

#### 2.2.2. ABTS Radical Scavenging Activity

The ABTS radical scavenging activity for BSE was analyzed following the methodology outlined in Sánchez-Moreno [29]. We used ascorbic acid as a standard material, and BSE was diluted using ethanol to concentrations of 0.5, 1, 2, and 4 mg/mL. Then, 7.4 mM ABTS and 2.6 mM potassium persulfate were mixed and incubated at room temperature for 16 h in the dark. The solution was diluted with ethanol to achieve an absorbance of 0.70 ± 0.02 at 734 nm, and then used for the experiment. The samples and final ABTS solution were mixed at a ratio of 1:9 and were then transferred into a 96-well plate. The plate was incubated at room temperature for 1 min in the dark, and the absorbance was measured at 734 nm using a microplate reader (Synergy 2, BioTek Instruments, Winooski, VT, USA).

#### 2.2.3. Total Polyphenol Content

The total polyphenol content for BSE was measured following the methodology outlined in Zhang et al. [30]. For the determination of the total polyphenol content, BSE was diluted to concentrations of 1, 2.5, 5, and 10 mg/mL. In a 96-well plate, 20 μL of BSE and 80 μL of DW were added, followed by the addition of 40 μL of Folin–Ciocalteu’s phenol reagent (Sigma-Aldrich, St. Louis, MO, USA). The mixture was allowed to react at room temperature for 3 min. Then, 60 μL of a 10% Na_2_CO_3_ solution was added, and the mixture was incubated at room temperature for 2 h. Absorbance was measured at 725 nm using a microplate reader (Synergy 2, BioTek Instruments, Winooski, VT, USA). Galic acid (DAEJUNG, Siheung, Republic of Korea) was used as the standard to create a calibration curve, which was then used to quantify the total polyphenol content of the sample.

### 2.3. Animal Experiments

**Figure 1:**
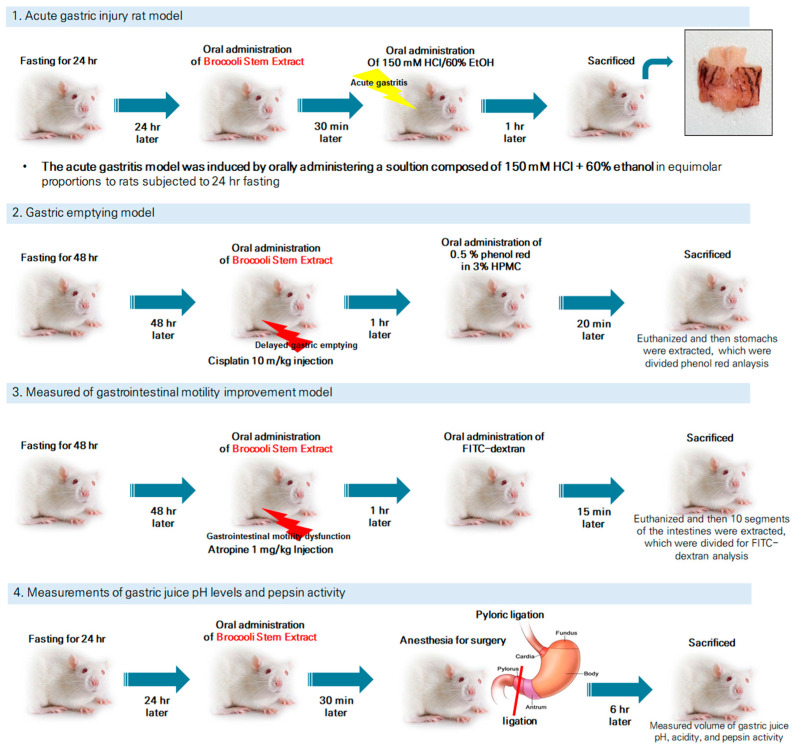
Summary of experimental design and methodology for acute gastritis induction, gastric emptying evaluation, gastrointestinal motility improvement, and gastric pH and pepsin activity measurements in SD rats. The lightning-shaped symbol indicates the damage inflicted on the rats.

Male Sprague Dawley (SD) rats (6 weeks old, 200–220 g) were purchased from Damool Sciences (Daejeon, Republic of Korea). The animals were housed under identical environmental conditions, including temperature (22 ± 2 °C), humidity (50–60%), and a 12 h light/dark cycle, to ensure uniform experimental settings. All rats used in this study were clinically healthy, with no signs of illness or abnormal behavior upon arrival. Animals showing signs of illness or significant weight variation outside the specified range were excluded. Before the experiments, all rats were fasted for 24 or 48 h. BSE was administered via oral gavage at doses of 50, 125, and 250 mg/kg. The control and model groups received an equal volume of distilled water (DW). Each experimental group consisted of six animals, based on the methodology described by Matsuda et al. (2013) [31]. All animal experiments were performed according to the guidelines of Jeonbuk National University (Jeonju, Republic of Korea) Institutional Animal Care and Use Committee (NON 2023-127). The study adhered to the ethical principles outlined in the EU Directive 2010/63/EU for the protection of animals used for scientific purposes. An overview of the experimental design is presented in Figure 1. Animals were randomly assigned to experimental groups using a computer-generated randomization list to ensure unbiased allocation. Randomization was performed prior to the start of the experiment to allocate the animals evenly across all groups (*n* = 6/group). To minimize bias, all experimental procedures and analyses were conducted by researchers blinded to the group assignments.

### 2.4. HCl/EtOH-Induced Acute Gastric Damage Animal Model

The 36 rats (*n* = 6/group) were randomly divided into the following six groups:

➢ Normal: Normal control group, only DW administration.
➢ Control: HCl/EtOH model group, administration of 150 mM HCl/60% ethanol and DW.
➢ MMSC: Positive control group, administration of 150 mM HCl/60% ethanol + Methyl Methionine Sulfonium Chloride 50 mg/kg.
➢ BSE groups: Experimental groups, administration of 150 mM HCl/60% ethanol + broccoli stem extracts (BSE) 50, 125, and 250 mg/kg (BSE 50, BSE 125, BSE 250, respectively).

The rats were fasting 24 h before animal experiments. The control and model groups were administered an equal volume of DW orally using a 22-gauge gavage needle (Jeungdo Bio & Plant, Seoul, Republic of Korea). MMSC was used as a reference for gastroprotective effects with anti-ulcer and gastric mucosal protection [32,33]. The BSE groups were treated with BSE extracts (50, 125, and 250 mg/kg) dissolved in DW. One hour after each substance administration, all rats were treated with 2 mL of 150 mM HCl/60% ethanol solution by oral administration. After 30 min, the rats were measured for clinical signs. The clinical symptom score was obtained following our previous study [34]. After one hour, the rats were anesthetized using isoflurane (Ifran soln, Hana Pharm Co., Ltd., Seoul, Republic of Korea), and blood was collected via the abdominal vein, followed by euthanasia using isoflurane. After confirming euthanasia, the stomach tissue was excised from the rats for subsequent analysis. The stomach was incised along the greater curvature and fixed in 1.5% paraformaldehyde (Sigma-Aldrich, St. Louis, MO, USA) for 15–30 min at room temperature. Fixed tissues were photographed, and the gastric damage area was determined with Image J software (version 1.8.0, National Institutes of Health, Bethesda, MD, USA).

Gastric lesion area (%) = (Gastric lesion/Total area) × 100

#### Evaluation of Clinical Signs

To evaluate the clinical signs of the experimental animals, a scoring system was used to quantify behavioral and physiological responses [34]. The scores were assigned as follows:

- 1 point: Normal walking and excited behavior;
- 5 points: Reduced movement compared to normal activity;
- 10 points: Slight movement observed only upon stimulation or minimal spontaneous activity;
- 15 points: Severe signs such as breathlessness and deep breathing.

All evaluations were conducted under blinded conditions to minimize potential bias. To ensure the objectivity of the scoring process, three assistant researchers, excluding the principal investigator, independently performed the evaluations in a blinded manner. The final scores were averaged to obtain consistent and reliable results.

### 2.5. Histological Analysis

To evaluate histological and microscopic scores, the stomachs were fixed in 10% formalin for 24 h and then embedded in paraffin to make paraffin blocks. The paraffin blocks were sectioned into 4 μm thick slices using a microtome and mounted on slide glasses for hematoxylin and eosin (H&E) staining. Each slide was observed under an optical microscope (Olympus, Tokyo, Japan), and images were captured using Olympus digital image software (analysis TS, Olympus Corp., Tokyo, Japan).

### 2.6. Macroscopic and Microscopic Evaluation of Stomach Tissues

The rats from which blood was collected were euthanized using isoflurane (Hana Pharm). After euthanasia, the stomach was removed, opened, and washed with PBS twice. The stomach was briefly fixed in a 10% formalin solution for 15 s for photography, then fixed again in buffered formalin for histology analysis. The macroscopic and microscopic scores for the damaged stomachs were obtained following Slimões et al. [1], as shown in Table 1.

### 2.7. ELISA Assay

Prostaglandin E2 (PGE2, UNEB0008, Assay Genie, Dublin, Ireland) concentration in gastric tissue was measured with an ELISA assay kit. The PGE2 concentration in the gastric tissue was measured according to the manufacturer’s protocol. The absorbance was measured at 450 nm using a microplate reader (Synergy 2, BioTek instrument, Chittenden County, VT, USA).

### 2.8. Quantitiative Real-Time PCR (qRT-PCR) Analysis

To measure inflammatory cytokine levels in stomach tissue, RNA was extracted using the RNeasy Mini Kit (74104, QIAGEN, Valencia, CA, USA). The extracted RNA was reverse transcribed into cDNA using the PrimeScript^™^ RT reagent with gDNA Eraser (RR047A, TaKaRa Bio Inc., Otsu, Japan). Subsequently, qRT-PCR was performed using the BioFACT^™^ 2X Real-Time PCR Master Mix, including SYBR^®^ Green I mixture, with high Rox reference dye (DQ385-40h, BIOFACT, Daejeon, Republic of Korea) on a two-step cycler. All expression levels were quantified by the ∆∆Ct method and normalized to GAPDH expression without bacterial groups. The bacterial groups were normalized to total bacteria. The primer sequences used for qPCR are shown in Table 2.

### 2.9. Western Blotting

Proteins from the rat stomach tissues were extracted using RIPA buffer, and their concentrations were determined using the BCA assay kit (23225, Thermo Scientific^™^, Middlesex County, MA, USA). The quantified proteins were separated by SDS-PAGE and then transferred onto a PVDF membrane. The membrane was blocked in 5% bovine serum albumin (BSA) to prevent nonspecific binding for 1 h at room temperature, followed by incubation with primary antibodies diluted 1:1000 in 5% BSA at 4 °C overnight. The antibodies used for analysis included the following: NF-κB (#8242, Cell Signaling, Danvers, MA, USA), p-NF-κB (#3033, Cell Signaling), IκBα (#4814, Cell Signaling), p-IκBα (#4814, Cell Signaling), and β-actin (#4970, Cell Signaling). The secondary antibodies, goat anti-rabbit IgG (#7074, Cell Signaling) or goat anti-mouse IgG (#7076, Cell Signaling), were diluted 1:2000 in 5% BSA and incubated for 2 h at room temperature. After antibody reaction, the membrane was washed with PBS-T, and protein bands were detected using an ECL solution (OP101-200, BIOFACT, Daejeon, Republic of Korea) with a Chemi-imager system (Alpha Innotech, San Leandro, CA, USA). The bands were quantified using Image J Software, and all results were normalized to the optimal density ration of β-actin.

### 2.10. Cisplatin-Induced Gastrointestinal Dysmotility Model

Cisplatin-induced gastrointestinal dysmotility was modeled by intraperitoneally injecting cisplatin (10 mg/kg) into the rats. The 36 rats (*n* = 6/group) were randomly divided into the following six groups:

➢ Normal: DW only;
➢ Control: Cisplatin i.p. injection + DW;
➢ Itopride: Cisplatin i.p. injection + itopride (30 mg/kg);
➢ BSE groups: Cisplatin i.p. injection + BSE (50, 125, 250 mg/kg).

Each substance was diluted in 3% hydroxyl propyl methyl cellulose (HPMC, Sigma-Aldrich, St. Louis, MO, USA) as a vehicle. One hour after cisplatin administration, a liquid meal containing 1.5% HPMC and 0.5% phenol red was orally administered. The percentage of gastric emptying was calculated from the following formula [35]:

Gastric emptying (%) = 1 − [(concentration of phenol red in test stomach)/(concentration of phenol red in standard stomach)] × 100

### 2.11. Measurement of Gastrointestinal Motility and Established Animal Model

The rats fasted for 48 h before the experiment. All 36 rats were randomly assigned into a total of 6 groups (*n* = 6/group).

➢ Normal: Normal control group, only DW administration.
➢ Control: Vehicle, atropine 1 mg/kg, negative control group.
➢ Mosapirde: Positive control group, atropine 1 mg/kg + Mosapride 10 mg/kg.
➢ BSE groups: Experimental groups, atropine 1 mg/kg + broccoli stems extracts (BSE) 50, 125, and 250 mg/kg (BSE 50, BSE 125, BSE 250, respectively).

Each substance was prepared with 3% HPMC as a vehicle, and atropine was dissolved in DW at a concentration of 1 mg/kg. The experimental groups (i.e., all except the normal group) were administered atropine intraperitoneally, while the normal group received an equal volume of DW. Then, 1 h after the administration of the substances, 1 mL/rats of FITC-dextran prepared at 6.25 mg/mL was administered orally to complete the modeling.

After administration of the substances, the rats were anesthetized and euthanized 15 m later to measure the amount of FITC-dextran remaining in the small intestine. The small intestine was excised, with the ends secured to prevent the contents from spilling out. The excised intestine was divided into 10 segments, and the geometric center (G.C.) was measured. Each segment was homogenized with 3 mL of a 0.05 M Tris buffer, then centrifuged at 800× *g* for 5 min. The supernatant was collected, and the fluorescent signal was measured using a microplate reader (Synergy, 2, BioTek instrument, Winooski, VT, USA) at 490–520 nm. The G.C. value was calculated using the following formula [36]:

G.C. = ∑(% of total fluorescent signal per segment × segment number)/100

### 2.12. Measurement of Anti-Secretory Activity in Pyloric Ligation

After 24 h of fasting, 30 rats were randomly assigned into five groups (*n* = 6/group).

➢ Control: Vehicle, negative control, pyloric ligation.
➢ MMSC: Positive control, Methyl Methionine Sulfonium Chloride 50 mg/kg, pyloric ligation.
➢ BSE groups: Experimental groups, administration of broccoli stem extracts (BSE) 50, 125, and 250 mg/kg (BSE 50, BSE 125, BSE 250, respectively), pyloric ligation.

Following the administration of the samples and 30 min after, the rats were anesthetized using isoflurane. An incision was made in the abdominal cavity, and pylori ligation was performed. After suturing the surgical site, the rats were placed back in their cages. Six h post-pyloric ligation, the rats were euthanized, and gastric juice was collected from the stomach. The supernatant of the gastric juice was obtained by centrifugation at 2000× *g* for 10 min. The volume and pH of the gastric juice were measured using a pH meter (Hanna, Woonsocket, RI, USA). Acidity was assessed by titration with 0.05 N NaOH using phenolphthalein as an indicator. Total acidity was calculated using the following formula [37]:

Total acidity (mEq/6h) = Vol. of gastric juice (mL) × Vol. of NaOH (mL) × normality of 0.05 N NaOH

Additionally, the supernatant of the gastric juice was utilized to measured pepsin activity. Pepsin activity was determined according to the following formula [38]:

Pepsin activity (Units/mL) = [(A_280_ sample − A_280_ blank) × dilution factor] ÷ t × v

t = Assay incubation time in min

v = volume of gastric juice (mL) added

### 2.13. Statistical Analysis

All results are expressed as mean±SEM (*n* = 6). The Shapiro–Wilk test was used to assess the normality of the data distribution. For datasets that met the assumption of normality, one-way ANOVA was conducted using the Prism 9.5 program (GraphPad Software, San Diego, CA, USA), followed by post hoc analysis using Tukey’s test. For datasets that did not meet normality, the Kruskal–Wallis test was performed as a non-parametric alternative. Statistical significance was determined at the *p* < 0.05 level.

## 3. Results

### 3.1. Antioxidant Activity of BSE

**Figure 2:**
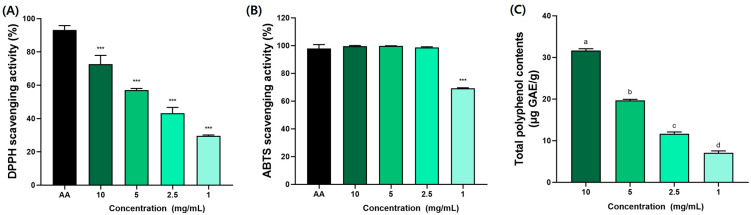
Antioxidant effects of broccoli stem extract (BSE) in (**A**) DPPH, (**B**) ABTS, and (**C**) total polyphenol contents. AA (ascorbic acid) was used as standard material. All results were acquired with third replicates to derive average value. Data were expressed as mean ± SEM (*n* = 6). *** *p* < 0.005 compared to AA group. ^a–d^ Different letters denote significant differences (*p* < 0.05) among groups, as determined by one-way ANOVA followed by Tukey’s multiple range test. Groups sharing at least one common letter (e.g., ‘a’ and ‘ab’) are not significantly different, whereas groups with no common letters (e.g., ‘a’ and ‘b’) are significantly different.

The antioxidant effects of BSE presented in Figure 2. The antioxidant activity of BSE measured in this study, with DPPH radical scavenging activity reaching 72.7 ± 3.7% at 10 mg/mL, aligns with findings from other studies on broccoli by-products. For instance, previous studies [39,40] reported that broccoli leaf extracts exhibited similar DPPH activity due to high levels of polyphenols and glucosinolates. Furthermore, the total polyphenol content of BSE (31.64 ± 0.26 µg GAE/g) was comparable. These findings support the hypothesis that the polyphenol and glucosinolate content of broccoli stems significantly contributes to their antioxidant potential.

### 3.2. Effects of BSE on Clinical Signs After HCl/EtOH-Induced Acute Gastric Injury

**Figure 3:**
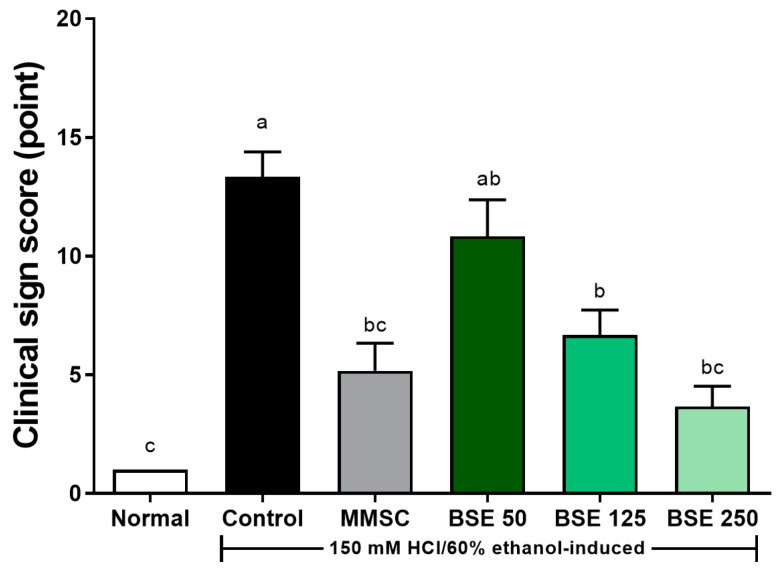
Effects of broccoli stem extract (BSE) on clinical sign scores, which evaluate behavioral and physiological responses, in acute gastric injury rat models. Normal, normal control group; Control, HCl/EtOH model group; MMSC, Methyl Methionine Sulfonium Chloride 50 mg/kg; BSE, broccoli stem extract 50, 125, and 250 mg/kg. Vertical bars represent mean ± SEM (*n* = 6). ^a–c^ Different letters denote significant differences (*p* < 0.05) among groups, as determined by one-way ANOVA followed by Tukey’s multiple range test. Groups sharing at least one common letter (e.g., ‘a’ and ‘ab’) are not significantly different, whereas groups with no common letters (e.g., ‘a’ and ‘b’) are significantly different.

The observed movements and clinical signs 30 min after administering the gastritis-induced solution (150 mM HCl/60% EtOH) to the rats with induced acute gastritis are presented in Figure 3. In the normal group, typical rat behavior was observed with no notable abnormalities. In the control group, the induction of acute gastritis led to a progressive decrease in movement, and the rats appeared to have increased difficulty breathing over time. In the groups treated with BSE, a dose-dependent alleviation of movement signs was observed compared to the control group. Notably, although movements were slower than those in the normal group, spontaneous movements were still evident in the BSE 250 group and the MMSC group.

### 3.3. Effects of BSE on HCl/EtOH-Induced Gastric Lesion and Mucosa Damage

**Figure 4:**
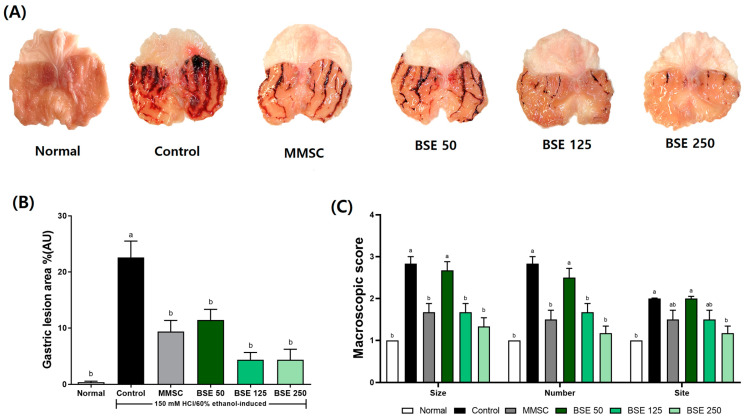
Effects of broccoli stem extract (BSE) on gastric surface protection and lesion severity in acute gastric injury rat models. (**A**) Gross appearance of stomach tissues; (**B**) ratios of hemorrhagic lesion area to total area; (**C**) macroscopic score. Normal, normal control group; Control, HCl/EtOH model group; MMSC, Methyl Methionine Sulfonium Chloride 50 mg/kg; BSE, broccoli stem extract 50, 125, and 250 mg/kg. Vertical bars represent mean ± SEM (*n* = 6). ^a,b^ Different letters denote significant differences (*p* < 0.05) among groups, as determined by one-way ANOVA followed by Tukey’s multiple range test. Groups sharing at least one common letter (e.g., ‘a’ and ‘ab’) are not significantly different, whereas groups with no common letters (e.g., ‘a’ and ‘b’) are significantly different.

The administration of the HCl/EtOH solution to the rats induced hemorrhagic ulcers in the glandular stomach, appearing as elongated black lines [4]. These hemorrhagic ulcers could be prevented in a dose-dependent manner with BSE pretreatment (Figure 4A). Additionally, the gastric lesion index was significantly reduced in all BSE-treated groups compared to the control group (Figure 4B). In terms of the macroscopic score for gastric damage, the BSE 125 and BSE 250 groups showed significant improvements in the size, number, and site of hemorrhagic ulcers compared to the control group (Figure 4C).

### 3.4. Effects of BSE on Histological Analysis in HCl/EtOH-Induced Rats

**Figure 5:**
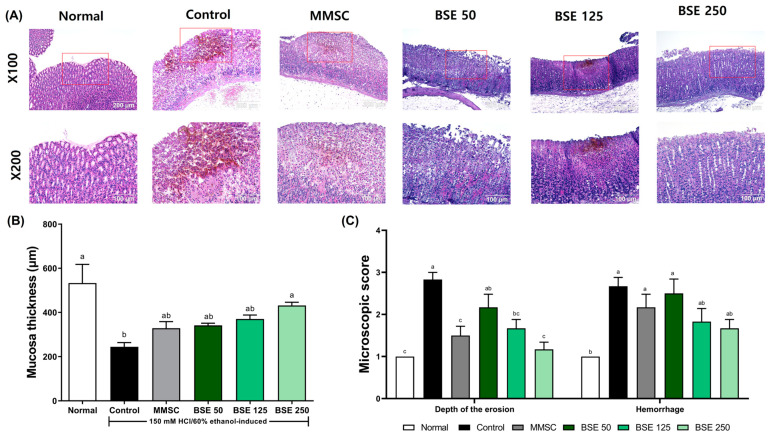
Effects of broccoli stem extract (BSE) on histological changes in gastric mucosa in acute gastric injury rat models. (**A**) Histological sections of gastric mucosa in rats. The red box in the X100 magnification image indicates the area shown in the ×200 magnification image; (**B**) mucosal thickness in rats; (**C**) microscopic score. Normal, normal control group; Control, HCl/EtOH model group; MMSC, Methyl Methionine Sulfonium Chloride 50 mg/kg; BSE, broccoli stem extract 50, 125, and 250 mg/kg. Vertical bars represent mean ± SEM (*n* = 6). ^a–c^ Different letters denote significant differences (*p* < 0.05) among groups, as determined by one-way ANOVA followed by Tukey’s multiple range test. Groups sharing at least one common letter (e.g., ‘a’ and ‘ab’) are not significantly different, whereas groups with no common letters (e.g., ‘a’ and ‘b’) are significantly different.

In Figure 5A, the control group exhibited severe superficial destruction of the gastric gland and significant hemorrhage following HCl/EtOH administration. Although hemorrhage was also observed in the MMSC group, the degree of recovery was greater compared to the control group. In the BSE 250 group, HCl/EtOH-induced gastric mucosal damage, including mucosal loss and hemorrhage, was significantly reduced. In addition, HCl/EtOH administration resulted in a decrease in mucosal thickness compared to the control group (Figure 5B). Pretreatment with BSE led to dose-dependent recovery of the reduced mucosal thickness, and microscopic scoring also revealed a reduction in the depth of erosion and hemorrhage score (Figure 5C).

### 3.5. Effects of BSE on NADPH Oxidase in HCl/EtOH-Induced Rats

**Figure 6:**
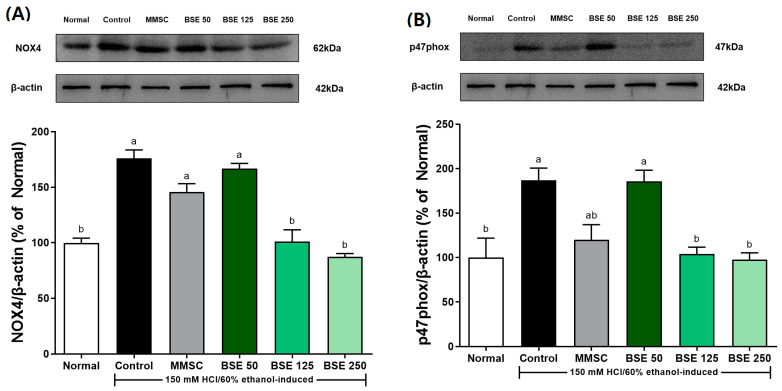
Effects of broccoli stem extract (BSE) on expression of NADPH oxidase expression of stomach tissue in acute gastric injury rat models. (**A**) NOX-4/β-actin; (**B**) p47phox/β-actin. Normal, normal control group; Control, HCl/EtOH model group; MMSC, Methyl Methionine Sulfonium Chloride 50 mg/kg; BSE, broccoli stem extract 50, 125, and 250 mg/kg. Vertical bars represent mean ± SEM (*n* = 6). ^a,b^ Different letters denote significant differences (*p* < 0.05) among groups, as determined by one-way ANOVA followed by Tukey’s multiple range test. Groups sharing at least one common letter (e.g., ‘a’ and ‘ab’) are not significantly different, whereas groups with no common letters (e.g., ‘a’ and ‘b’) are significantly different.

The administration of HCl/EtOH increased the expression of NOX4 in gastric tissue in the control group compared to the normal group (Figure 6A). This response was prevented in the BSE 125 and BSE 250 groups. Similarly, HCl/EtOH intake increased the expression of p47 phox in the control group (Figure 6B), while the BSE 125 and BSE 250 groups showed prevention of this increase to levels comparable to the normal group.

### 3.6. Effects of BSE on NF-κB Signaling Pathway in HCl/EtOH-Induced Rats

**Figure 7:**
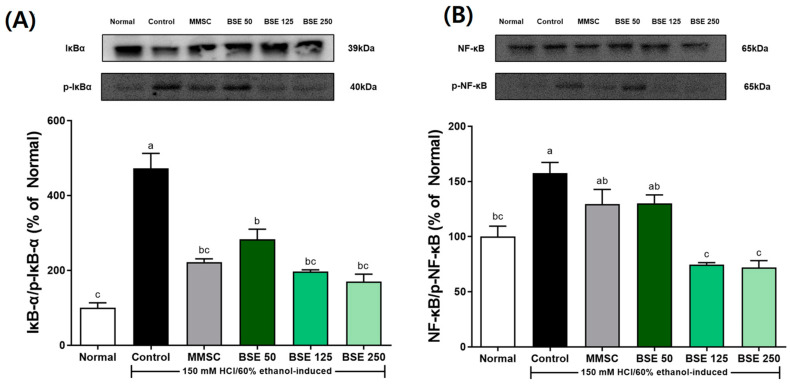
Effects of broccoli stem extract (BSE) on expression of NF-κB pathway components stomach tissue in acute gastric injury rat models. (**A**) IκB-α/p-IκB-α; (**B**) NF-κB/p-NF-κB. Normal, normal control group; Control, HCl/EtOH model group; MMSC, Methyl Methionine Sulfonium Chloride 50 mg/kg; BSE, broccoli stem extract 50, 125, and 250 mg/kg. Vertical bars represent mean ± SEM (*n* = 6). ^a–c^ Different letters denote significant differences (*p* < 0.05) among groups, as determined by one-way ANOVA followed by Tukey’s multiple range test. Groups sharing at least one common letter (e.g., ‘a’ and ‘ab’) are not significantly different, whereas groups with no common letters (e.g., ‘a’ and ‘b’) are significantly different.

We investigated the effects of BSC on the NF-κB signaling pathway and inflammatory cytokine levels by measuring the protein and mRNA expression of stomach tissues. As shown in Figure 7A,B, the expression of p-IκBα/IκBα was significantly increased in the control group compared to the normal group. Additionally, the expression of p-NF-κB/NF-κB also increased in the control group. This indicated that the administration of HCl/ETOH led to the activation of the NF-κB signaling pathway. However, pretreatment with BSE decreased the expression of p-IκBα/IκBα and p-NF-κB/NF-κB. In particular, the BSE 250 group showed a significant decrease in p-IκBα/IκBα and p-NF-κB/NF-κB expression compared to the control group.

**Figure 8:**
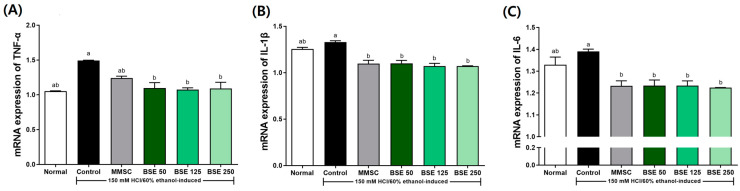
Effects of broccoli stem extract (BSE) on pro-inflammatory cytokine mRNA expression of stomach tissue in acute gastric injury rat models. (**A**) TNF-α/GAPDH; (**B**) IL-1β/GAPDH; (**C**) IL-6/GAPDH. Normal, normal control group; Control, HCl/EtOH model group; MMSC Methyl Methionine Sulfonium Chloride 50 mg/kg; BSE, broccoli stem extract 50, 125, and 250 mg/kg. Vertical bars represent mean ± SEM (*n* = 6). ^a,b^ Different letters denote significant differences (*p* < 0.05) among groups, as determined by one-way ANOVA followed by Tukey’s multiple range test. Groups sharing at least one common letter (e.g., ‘a’ and ‘ab’) are not significantly different, whereas groups with no common letters (e.g., ‘a’ and ‘b’) are significantly different.

Additionally, the expression of inflammatory cytokines induced by acute gastric injury from HCl/EtOH administration was examined (Figure 8). In the control group, the expression of TNF-α, IL-1β, and IL-6 was slightly increased compared to the normal group. This elevated expression was significantly reduced by pretreatment with BSE compared to the control group. This trend was similar to the results observed in the positive control group treated with MMSC.

**Figure 9:**
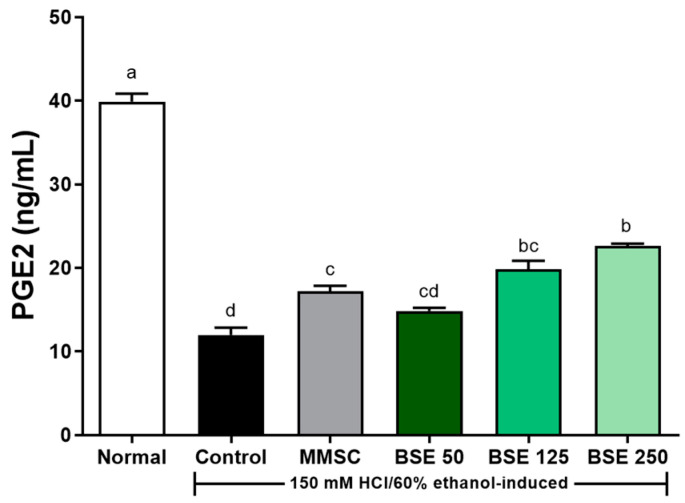
Effects of BSE on PGE_2_ levels of stomach tissue in acute gastric injury rat model. Normal, normal control group; Control, HCl/EtOH model group; MMSC, Methyl Methionine Sulfonium Chloride 50 mg/kg; BSE, broccoli stem extract 50, 125, and 250 mg/kg. Vertical bars represent mean ± SEM (*n* = 6). ^a–d^ Different letters denote significant differences (*p* < 0.05) among groups, as determined by one-way ANOVA followed by Tukey’s multiple range test. Groups sharing at least one common letter (e.g., ‘b’ and ‘bc’) are not significantly different, whereas groups with no common letters (e.g., ‘a’ and ‘b’) are significantly different.

The concentration of PGE_2_ in gastric tissue was significantly reduced in the control group compared to the normal group (*p* < 0.0001, Figure 9). PGE_2_ plays an important role as a mediator of inflammatory response by inhibiting inflammatory cytokines such as TNF-α and IL-β. The reduced concentration of PGE_2_ induced by HCl/EtOH administration increased in a dose-dependent manner with BSE pretreatment. Significant increases in PGE_2_ concentration were observed in the BSE 125 and BSE 250 groups compared to the control group (*p* < 0.0001). Therefore, these results suggest that BSE pretreatment may help reduce the risk of acute gastritis by inhibiting the NF-κB signaling pathway, which is activated during acute gastritis, and by inducing the production of PGE_2_, a regulator of immune and inflammatory responses, thereby suppressing inflammatory cytokines such as TNF-α and IL-1β.

### 3.7. Effects of BSE on Gastric Emptying in Cisplatin-Induced Rats

**Figure 10:**
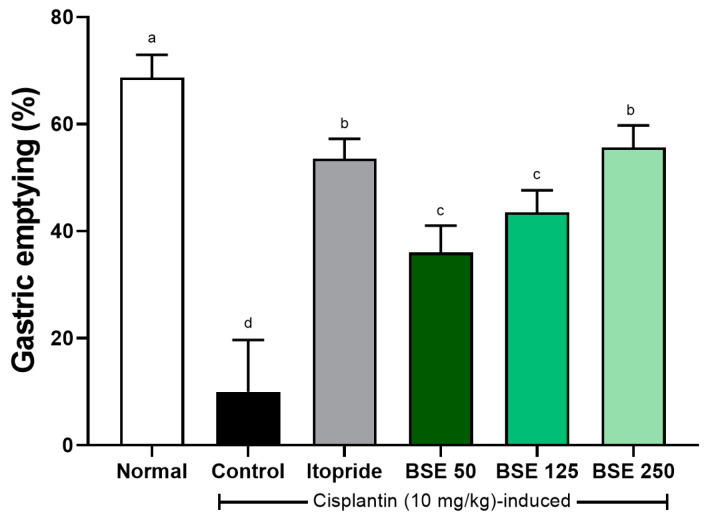
Effects of BSE on gastric emptying in cisplatin-induced SD rats. Gastric emptying was measured as percentage of food emptied from stomach after test meal. Normal, normal control group; Control, cisplatin only group; Itopride, itopride 30 mg/kg; BSE, broccoli stem extract 50, 125, and 250 mg/kg. Vertical bars represent mean ± SEM (*n* = 6). ^a–d^ Different letters denote significant differences (*p* < 0.05) among groups, as determined by one-way ANOVA followed by Tukey’s multiple range test. Groups sharing at least one common letter (e.g., ‘a’ and ‘ab’) are not significantly different, whereas groups with no common letters (e.g., ‘a’ and ‘b’) are significantly different.

Cisplatin increased gastric retention and induced delayed gastric emptying in rats [41]. Gastric emptying capacity, which was 68.7 ± 1.8% in the normal group, significantly decreased to 10.0 ± 4.0% in the control group (Figure 10). The reduced gastric emptying capacity increased in a dose-dependent manner with BSE administration (50, 125, and 250 mg/kg), resulting in 36.0 ± 2.1%, 43.5 ± 1.7%, and 55.7 ± 1.7%, respectively.

### 3.8. Effects of BSE on Gastrointestinal Motility in Atropine-Induced Rats

**Figure 11:**
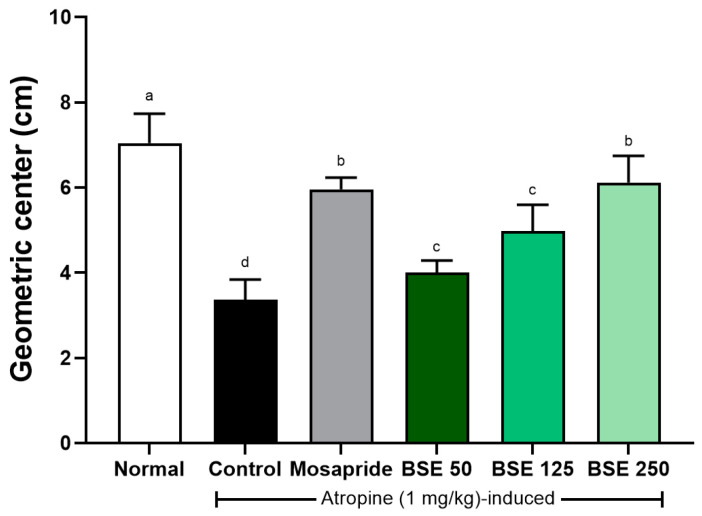
Effects of broccoli stem extract (BSE) on intestinal motility in atropine-induced gastrointestinal dysmotility in SD rats. Intestinal motility was assessed using geometric center method, which measures transit of marker dye through intestine. Normal, normal control group; Control, atropine control group; Mosapride, mosapride 10 mg/kg; BSE, broccoli stem extract 50, 125, and 250 mg/kg. Vertical bars represent mean ± SEM (*n* = 6). ^a–d^ Different letters denote significant differences (*p* < 0.05) among groups, as determined by one-way ANOVA followed by Tukey’s multiple range test. Groups sharing at least one common letter (e.g., ‘a’ and ‘ab’) are not significantly different, whereas groups with no common letters (e.g., ‘a’ and ‘b’) are significantly different.

Atropine is an anticholinergic agent that binds to acetylcholine receptors, blocking the effects of acetylcholine [42]. Although several studies have indicated that atropine functions as an antispasmodic analgesic, incorrect use can reduce intestinal motility in patients with gastric ulcers, potentially exacerbating gastrointestinal symptoms [43]. In our study, rats administered atropine (control group) exhibited a decreased geometric center compared to the normal group (Figure 11). This decrease tended to recover in a dose-dependent manner with BSE. These results suggest that the delayed gastric motility induced by atropine can be restored though BSE administration.

### 3.9. Effects of BSE on Gastric Secretion in Pyloric Ligation Rat Model

**Figure 12:**
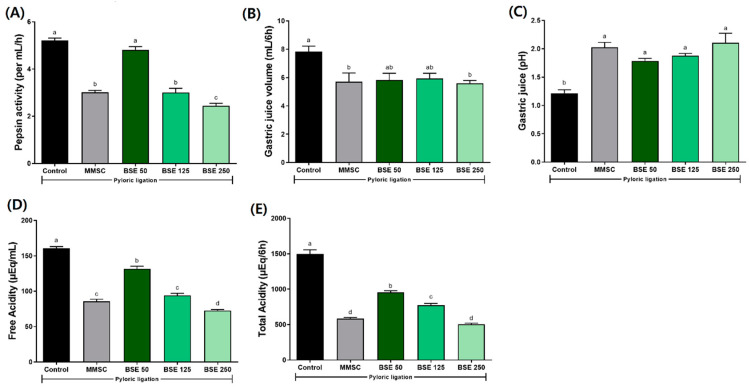
Effects of BSE on gastric secretion parameters in pyloric ligation rats. (**A**) Pepsin activity (per mL/h); (**B**) gastric juice volume (mL/6h); (**C**) gastric juice (pH); (**D**) free acidity (μEq/mL); (**E**) total acidity (μEq/6h). Control, pyloric ligation model group; MMSC, Methyl Methionine Sulfonium Chloride 50 mg/kg; BSE, broccoli stem extract 50, 125, and 250 mg/kg. Vertical bars represent mean ± SEM (*n* = 6). ^a–c^ Different letters denote significant differences (*p* < 0.05) among groups, as determined by one-way ANOVA followed by Tukey’s multiple range test. Groups sharing at least one common letter (e.g., ‘a’ and ‘ab’) are not significantly different, whereas groups with no common letters (e.g., ‘a’ and ‘b’) are significantly different.

To investigate the mechanism affecting gastric acid secretion, we performed pyloric ligation surgery on rats and evaluated the related parameters. Prior to pylorus ligation, rats were pretreated with BSE at three different concentrations. Results showed that in the BSE 125 and 250 groups, pepsin activity, gastric juice volume, free acidity, and total acidity were significantly reduced compared to the control group (Figure 12). These findings suggest that BSE has potential gastroprotective effects through its gastric protective actions.

## 4. Discussion

This study aimed to investigate the gastroprotective effects of BSE using the HCl/EtOH-induced gastritis model. Acute gastritis, often associated with excessive alcohol consumption, causes significant tissue damage in the gastric mucosa and remains a major global health concern [44,45,46,47]. Ethanol, particularly when combined with HCl, exacerbates gastric mucosal damage by increasing gastric acid and pepsin secretion, leading to severe tissue injury and inflammation [38,47]. This model was employed to evaluate the potential of BSE as a gastroprotective agent and its ability to mitigate ethanol-induced gastric lesions.

The gastroprotective effects of BSE observed in this study may be attributed to its rich content of bioactive compounds, particularly glucosinolates, sulforaphane, and polyphenols. Previous studies have demonstrated that sulforaphane, a key metabolite of glucoraphanin found in broccoli, exerts potent antioxidant and anti-inflammatory effects by modulating the NF-κB signaling pathway and enhancing phase II detoxification enzymes [48,49]. Additionally, flavonoids such as quercetin and kaempferol, which are abundant in broccoli leaves and stems, were shown to contribute to radical scavenging activity and reduce oxidative stress [49].

MMSC (Methyl Methionine Sulfonium Chloride) was selected as a positive control in this study due to its established gastroprotective properties in experimental models of gastric injury. Previous studies have demonstrated that MMSC exhibits anti-ulcer and gastric mucosal protective effects through various mechanisms [32,33]. These properties make it an appropriate comparator for evaluating the gastroprotective effects of BSE. Furthermore, the bioactive compounds in broccoli stems, such as glucosinolates and sulforaphane, share similar protective mechanisms with MMSC in mitigating gastric mucosal injury. This parallel highlights the relevance of MMSC as a positive control for assessing the efficacy of BSE in protecting against acute gastric injury. Although this study primarily quantified total polyphenol content, future work should include a detailed analysis of specific bioactive compounds to further elucidate the molecular mechanisms underlying BSE’s gastroprotective effects.

Damage to the gastric tissue caused by HCl/EtOH leads to hemorrhage, which reduces blood flow. This results in the increased secretion of gastric acid due to the expulsion of Na^+^ and influx of K^+^, leading to a decrease in gastric juice pH and exacerbating tissue damage [50]. In our study, we observed gastric tissue damage and hemorrhage following HCl/EtOH administration (Figure 4A) and found that pretreatment with BSE prevented such tissue damage. Additionally, we established a pylorus ligation model to further investigate the effect of BSE on gastric juice pH [51]. Our results showed that BSE pretreatment reduced total acidity and gastric juice volume, and also increased the pH of gastric juice (Figure 12). These findings indicate that BSE possesses anti-secretory activity, supporting its effectiveness in alleviating gastric tissue damage in rats with acute gastritis induced by HCl/EtOH.

However, the HCl/EtOH-induced gastric lesion model has certain limitations. It primarily replicates acute gastric mucosal injury and does not fully reflect the chronic progression of gastritis observed in humans. Additionally, interspecies differences between rats and humans, particularly in gastric physiology and response to treatment, should be considered when interpreting these findings. Despite these limitations, this model is widely accepted due to its reproducibility, simplicity, and ability to consistently induce gastric lesions for evaluating potential therapeutic agents.

Our study demonstrated that oxidative stress and inflammation, as indicated by the upregulation of NOX-4, play a significant role in the pathogenesis of HCl/EtOH-induced gastric injury (Figure 6A). NOX-4, a key member of the ROS-generating NADPH oxidase family, was significantly elevated in the gastric tissue of HCl/EtOH-treated rats, as is consistent with its established role in oxidative stress-mediated tissue damage [52,53]. Importantly, BSE pretreatment markedly reduced NOX-4 expression, suggesting its potential to mitigate oxidative stress and inhibit ROS production. These findings align with previous studies demonstrating that NOX-4 downregulation alleviates gastric mucosal injury and apoptosis by suppressing caspase-3 activation [52]. Furthermore, BSE may exert its effects by modulating signaling pathways, such as NF-κB and MAPK, which are known to regulate NADPH oxidase activity [53]. Thus, the downregulation of NOX-4 by BSE may be a key mechanism underlying its gastroprotective effects.

The dysregulation of the NF-κB pathway contributes to the onset of inflammation in epithelial and immune cells, and if this persists, it can increase the risk or chronic inflammation of gastric cancer [54]. The NF-κB pathway is activated by ROS generated within cells [55]. Once activated, the NF-κB pathway increases the expression of inflammatory cytokines such as TNF-α and IL-1β [54]. The primary mechanism of NF-κB activation involves the degradation of IκB-α; phosphorylated IκBα undergoes ubiquitination through downstream mechanisms, resulting in the release of NF-κB from the cytoplasm, its translocation to the nucleus, and subsequent regulation of downstream gene transcription [54]. Since NF-κB induces both acute and chronic inflammation, research on gastrointestinal inflammation often focuses on regulating this signaling pathway. In our study, we also confirmed the activation of NF-κB and IκB (Figure 7) and we found that phosphorylated IκB-α was effectively inhibited by BSE (Figure 7B). Other studies have concluded that downregulating the increased expression of NF-κB due to acute gastritis is beneficial for improving gastritis conditions [56,57]. BSE is hypothesized to inhibit NF-κB activation by modulating upstream signaling pathways, such as IκBα phosphorylation. This inhibition likely reduces the transcription of pro-inflammatory cytokines, including TNF-α and IL-6, which are key mediators of gastric inflammation [56,57]. The bioactive compounds in BSE, such as sulforaphane and polyphenols, are known to exhibit these inhibitory effects through direct interaction with NF-κB signaling components. Although this study focused on NF-κB signaling and cytokine modulation, future research should incorporate assays measuring oxidative stress markers, such as MDA, SOD, and GSH, to provide a comprehensive understanding of BSE’s gastroprotective mechanisms. Additionally, apoptosis-related proteins, such as Bax, Bcl-2, and Caspase-3, could be analyzed to elucidate BSE’s role in preventing gastric mucosal cell death.

Our study demonstrated that BSE significantly reduced the production of pro-inflammatory cytokines, including TNF-α, IL-1β, and IL-6, in HCl/EtOH-induced gastric injury (Figure 8). These findings indicate the anti-inflammatory potential of BSE in mitigating gastric mucosal damage. TNF-α and IL-6 are key mediators of gastric inflammation, with TNF-α initiating acute inflammatory responses and delaying ulcer healing by inhibiting angiogenesis and cell proliferation [58,59]. Similarly, IL-6 plays a critical role in both acute and chronic inflammation by activating T cells and B cells [60]. Previous studies have shown that broccoli extract reduces inflammatory cytokine production in various models, including colon cancer [61] and macrophage activation induced by sulforaphane [62]. These results align with our findings, highlighting the potential of BSE as a natural therapeutic agent for controlling inflammation in gastric mucosal injury.

Our study demonstrated that BSE restored the reduced PGE_2_ levels in gastric tissue following HCl/EtOH-induced injury. PGE_2_ is a key mediator of gastric mucosal defense, playing a critical role in reducing gastric acid secretion, enhancing mucus layer thickness, and improving mucosal blood flow [4,45]. A previous study has shown that HCl/EtOH administration significantly decreases PGE_2_ levels, leading to gastric ulcers and hemorrhage [4]. The restoration of PGE_2_ levels by BSE pretreatment suggests that BSE enhances gastric defense mechanisms, inhibits NF-κB pathway activation, and mitigates gastric mucosal damage.

Immune dysregulation in the stomach can directly and indirectly affect gastric motility [63]. When gastric motility disorders or gastrointestinal dysfunction occur, symptoms such as nausea, vomiting, and abdominal pain can significantly reduce patients’ quality of life [64]. Furthermore, these gastrointestinal disorders are associated with autonomic–nervous system and enteric neurons, potentially leading to complications in metabolic diseases such as diabetes [65]. In this study, we evaluated the effects of BSE on gastrointestinal motility in the presence of cisplatin, a drug affecting 5HT_3_ receptors, and atropine, an anticholinergic agent (Figure 11 and Figure 12). BSE improved delayed gastric emptying and intestinal motility induced by both drugs. However, this study only assessed gastric emptying and gastrointestinal motility, and further research is needed to elucidate the underlying mechanisms.

Additionally, the specific bioactive compounds within BSE responsible for these effects were not identified, and the molecular mechanisms regulating its gastroprotective properties remain unexplored. While this study demonstrates significant effects in a rat model, the applicability of these findings to humans requires further validation through clinical trials. Furthermore, the long-term safety and efficacy of BSE administration were not assessed in this study, warranting additional research. Future studies should focus on identifying these compounds and investigating their interaction with key pathways involved in gastric inflammation, oxidative stress, and motility regulation. These efforts will provide a more comprehensive understanding of the therapeutic potential of BSE.

## 5. Conclusions

In conclusion, we demonstrated the gastroprotective effects and enhanced gastrointestinal motility of BSE in a HCl/EtOH-induced acute gastritis rat model. BSE exhibited strong antioxidant activity, which contributed to its anti-inflammatory effects through the inhibition of the NF-κB pathway and the enhancement of gastric mucosal PGE_2_ synthesis, establishing its protective role in the stomach. Additionally, BSE administration increased gastrointestinal motility, suggesting a link to the suppression of gastric acid secretion.

This study also provides novel insights into the application of broccoli by-products, particularly the stems, as a natural and sustainable source for promoting gastric health. While the observed effects suggest potential for the prevention of acute gastritis, further studies are needed to identify specific bioactive compounds in BSE, such as glucosinolates and polyphenols, and to validate its therapeutic potential through human clinical trials. Based on these findings, BSE shows promise as a valuable agent for the prevention of acute gastritis and the promotion of gastric health.

## Figures and Tables

**Table 1:** Macroscopic and microscopic score evaluations in rat stomach tissues.

Macroscopic			
Hemorrhage	Score 1	Score 2	Score 3
Size	Punctiform (focal < 2 mm)	Mild (2–5 mm)	Intense or in band (<5 mm)
Number	0–4	5–6	≥7
Site	Unilaterals	Bilaterals	
**Microscopic**			
Score	1	2	3
Depth of the erosion	Up to 1/3 of total mucosa depth	Up to 1/3 of total mucosa depth	Total mucosa
Hemorrhage	Focal	Mild	Severe

**Table 2:** qRT-PCR primer sequences.

Gene Name	Sequence of PCR Primer (5′-3′)	
TNF-α	F	TGATCCGAGATGTGGAACTG
	R	CGAGCAGGAGTAAGAAGAGG
IL-1β	F	TGACCCATGTGAGCTGAAAG
	R	GGGATTTTGTCGTTGCTTGT
IL-6	F	CCGGAGAGGAGACTTCACAG
	R	CCATAGTGCAGGAGCGTACAGT
GAPDH	F	TGACCTCAACTACATGGTCTACA
	R	CTTCCCATTCTCGGCCTTG

qRT-PCR, quantitative reverse transcription polymerase chain reaction; TNF-α, tumor necrosis factor alpha; IL-1β, interleukin-1 beta; IL-6, interleukin-6; GAPDH, glyceraldehyde-3-phosphate dehydrogenase (housekeeping gene). qRT-PCR was performed to quantify expression of inflammatory cytokines (TNF-α, IL-1β, and IL-6) and housekeeping gene GAPDH in gastric tissue.

## Data Availability

The data presented in this study are available on request from the corresponding author. The data are not publicly available due to ethical restrictions.
